# Unraveling the COVID-19 Severity Hubs and Interplays in Inflammatory-Related RNA–Protein Networks

**DOI:** 10.3390/ijms26094412

**Published:** 2025-05-06

**Authors:** Heewon Park, Qingbo S. Wang, Takanori Hasegawa, Ho Namkoong, Hiroko Tanaka, Ryuji Koike, Yuko Kitagawa, Akinori Kimura, Seiya Imoto, Takanori Kanai, Koichi Fukunaga, Seishi Ogawa, Yukinori Okada, Satoru Miyano

**Affiliations:** 1 School of Mathematics Statistics and Data Science, Sungshin Women’s University, Seoul 02844, Republic of Korea; heewonn.park@gmail.com; 2 M&D Data Science Center, Institute of Science Tokyo, Tokyo 113-8510, Japan; 3 Human Genome Center, Institute of Medical Science, University of Tokyo, 4-6-1 Shirokane-dai, Minato-ku, Tokyo 108-8639, Japan; 4 Department of Genome Informatics, Graduate School of Medicine, The University of Tokyo, Tokyo 113-8654, Japan; 5 Department of Statistical Genetics, Osaka University Graduate School of Medicine, Suita 565-0871, Japan; 6 Department of Infectious Diseases, Keio University School of Medicine, Tokyo 160-8582, Japan; 7 Health Science Research and Development Center (HeRD), Tokyo Medical and Dental University, Tokyo 113-8510, Japan; 8 Department of Surgery, Keio University School of Medicine, Tokyo 160-8582, Japan; 9 Medical Research Institute, Tokyo Medical and Dental University, Tokyo 113-8510, Japan; 10 Division of Gastroenterology and Hepatology, Department of Internal Medicine, Keio University School of Medicine, 35 Shinanomachi, Shinjuku, Tokyo 160-8582, Japan; 11 Division of Pulmonary Medicine, Department of Internal Medicine, Keio University School of Medicine, Tokyo 160-8582, Japan; 12 Department of Pathology and Tumor Biology, Graduate School of Medicine, Kyoto University, Tokyo 606-8507, Japan; 13 Institute for the Advanced Study of Human Biology (WPI-ASHBi), Kyoto University, Kyoto 606-8501, Japan

**Keywords:** COVID-19, severity, RNA–protein networks, immunogenes, ACKR2

## Abstract

The rapid worldwide transmission of coronavirus disease 2019 (COVID-19), caused by severe acute respiratory syndrome coronavirus 2 (SARS-CoV-2), has led to severe cases of hypoxia, acute respiratory distress syndrome, multi-organ failure, and ultimately death. Small-scale molecular interactions have been analyzed by focusing on several genes/single genes, providing important insights; however, genome-wide multi-omics comprehensive molecular interactions have not yet been well investigated with the exception of GWAS and eQTLm, both of which show genetic risks. From April of 2020 until now, we have created a Japan-wide system, initially named the Japan COVID-19 Task Force. This system has collected more than 6500 COVID-19 patients’ peripheral blood and as much associated clinical information as possible from a network of more than 120 hospitals. DNA, RNA, serum, and plasma were extracted and stored in this bank. This study unravels the interplay of inflammatory gene networks that induce different COVID-19 severity levels (mild, moderate, severe, and critical) by using multi-omics data from the Japan COVID-19 Task Force. We analyze RNA and protein expressions to estimate severity-specific inflammation networks that uncover the interplay between RNA and protein networks via ligand–receptor pairs. Our large-scale RNA/protein expression data analysis reveals that the atypical chemokine receptor 2 (ACKR2) acts as a key broker linking RNA and protein inflammation networks to induce COVID-19 critical severity. ACKR2 emerges in RNA and protein inflammation networks, showing active interplay in high-severity cases and weak interactions in mild cases. The results also show severity-specific molecular interactions between interleukin (IL), cytokine receptor activity, cell adhesion, and interactions involving the CC chemokine ligand (CCL) gene family and ACKR2.

## 1. Introduction

Uncovering the inflammatory pathways associated with COVID-19 pathogenesis, including induction, function, downstream signaling, and inflammatory cytokines, is critical for developing effective COVID-19 therapeutic strategies [1]. Controlling inflammation is a promising approach to managing COVID-19 [2]. Several studies have been conducted to elucidate the inflammatory mechanisms involved in COVID-19 infection. Lee et al. [3] identified the therapeutic targets for severe inflammation in COVID-19 patients by analyzing single-cell RNA sequencing data. By clustering gene expression data, they inferred immune cell composition according to COVID-19 severity and analyzed the expression levels of imaging target molecules related to inflammation in macrophage clusters using single-cell RNA sequencing data. Additionally, Lee et al. [3] filtered potential therapeutic targets by identifying targetable molecules that are enriched in severe COVID-19 cases associated with hyperinflammation. Xu et al. [4] performed single-cell transcriptome data analysis and calculated single-cell pyroptosis signature scoring based on the difference in expression levels of pyroptosis marker genes. They then compared the pyroptosis scores between cell groups to characterize the pyroptotic states of cell groups and demonstrated that the inflammatory type of programmed cell death plays a critical role in the SARS-CoV-2-induced cytokine storm. A genome-wide association study of a SARS-CoV-2-negative cohort revealed that genetic risk of severe COVID-19 is correlated with low inflammatory marker levels [5]. Another genome-wide association study identified a variant close to the ‘dedicator of cytokinesis 2’ gene (DOCK2) from 2393 cases of COVID-19 in a cohort of Japanese individuals with 3289 unaffected controls from the Japan COVID-19 Task Force [6]. However, this allele is common only in East Asian individuals, and is rare in Europeans. Namkoong et al. [6] performed a genome-wide association study. They identified a genetic variant on chromosome 5 at 5q35 located near the DOCK2 gene, which was significantly associated with severe COVID-19 in individuals younger than 65. Bulk RNA sequencing of peripheral blood samples demonstrated that DOCK2 expression was downregulated in severe cases. Furthermore, single-cell RNA sequencing revealed cell type-specific suppression of DOCK2, with the risk allele exerting a COVID-19-specific inhibitory effect on DOCK2 expression in non-classical monocytes.

Lourda et al. [7] identified specific immunotypes with potential predictive value for key clinical features associated with COVID-19. In contrast to mild cases, patients with severe COVID-19 display heightened TNF/IL-1β-driven inflammation across peripheral blood mononuclear cells. Furthermore, severe cases uniquely demonstrate coactivated type I interferon responses in classical monocytes [8]. Metatranscriptomic sequencing of bronchoalveolar lavage fluid from eight COVID-19 cases revealed elevated proinflammatory gene expression, particularly for chemokines. Indications include hypercytokinemia and robust ISG induction with immunopathogenic potential along with increases in activated dendritic cells and neutrophils [9]. Currently, the COVID-19 Host Genetics Initiative has continued to update the human genetic architecture of COVID-19 and to contribute to worldwide knowledge of COVID-19 host genetics [10]. Large-scale studies are currently ongoing. The Japan COVID-19 Task Force has also conducted eQTL analysis. This analysis investigated whole-blood RNA-seq data from 465 genotyped samples from 359 severe and 106 non-severe cases for use in drawing the transcriptional regulation landscape [11]. Wang et al. [11] conducted differential gene expression analysis to identify genes with elevated expression in severe COVID-19 cases, finding that these are predominantly associated with innate immune functions. Additionally, they assessed the modest yet significant impact of COVID-19 phenotype on eQTL discovery and highlighted the presence of severity-associated interaction eQTLs (ieQTLs). One of the unexplored remaining tasks in this project is to build a landscape of comprehensive gene networks which interact with the protein networks that induce COVID-19 severity.

Although many studies have investigated the inflammation mechanism in COVID-19 and identified related markers based on single-gene abnormalities, relatively little attention has been paid to the molecular interplay between crucial markers. Analysis based on single genes is not sufficient to uncover COVID-19 mechanisms, as these complex mechanisms involve several genes that are intricately connected in a molecular network rather than perturbation of a single gene. We aim to find a clearer therapeutic target with the comprehensive network map.

This study addresses one of the next challenges of the Japan COVID-19 Task Force. The aim is to identify inflammatory gene networks that can characterize COVID-19 severity as mild, moderate, severe, and critical by using multi-omics data from the Japan COVID-19 Task Force. We analyzed whole-blood RNA-seq data from 1102 genotyped samples and protein expression data from 1384 genotyped samples.

We considered the 839 inflammatory response genes involved in the gene ontology (GO) terms for inflammatory response (GO:0006954), then estimated a severity-specific gene network using RNA and protein expression data on the four COVID-19 severity categories. The estimated gene regulatory networks based on RNA and protein expression of the inflammatory response genes are called the RNA inflammation network and protein inflammation network, respectively. RNA networks are informative for studying gene expression and regulation, but are insufficient for understanding disease-related protein functions. Thus, we aim to investigate RNA inflammatory networks as well as the broader RNA–protein crosstalk in the context of COVID-19. We focus on the interplay between RNA and protein inflammation networks, revealing the interactions between the two networks based on ligand–recipient pairs. This study can provide a deeper and more comprehensive understanding of protein–protein interaction networks that underlie diseases such as COVID-19. Although many studies have been conducted to uncover COVID-19 mechanisms based on Asian COVID-19 genotyped samples, lack of samples has been recognized as a limitation in these studies (i.e., 95 samples in South Asia [12], 386 samples in East Asian and South Asian ancestries [13], 332 samples in Chinese [14], 1072 samples of Chinese ancestry [15], etc.). To the best of our knowledge, this is the first large-scale study to perform multi-omics data analysis of gene networks for Asian COVID-19 genotyped samples.

**Figure 1:**
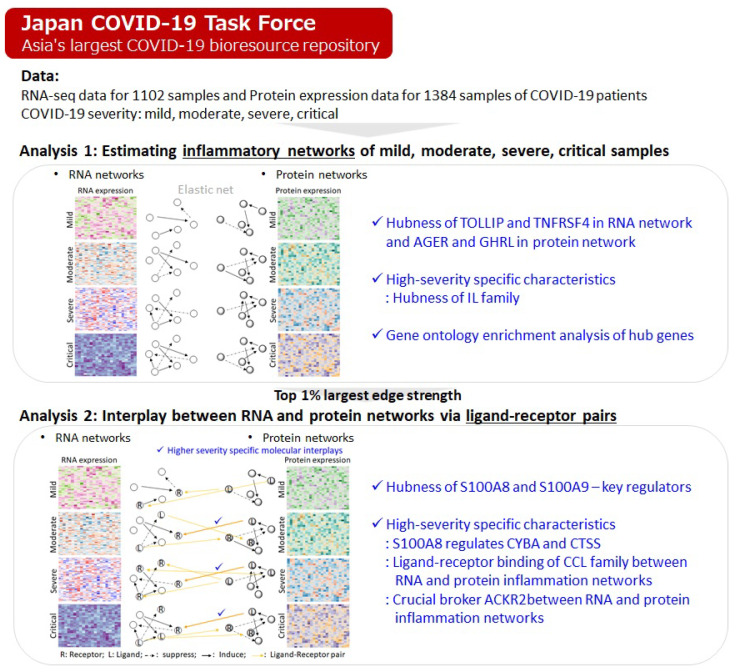
Overview of this study, including inflammatory network analysis via RNA and protein inflammation networks. Based on four levels of COVID-19 severity (mild, moderate, severe, critical), we first estimate RNA and protein networks based on the respective expression levels of RNA and protein (Analysis 1). Then, the interplays between RNA and protein networks are estimated using ligand–receptor pairs.

Figure 1 presents an overview of the inflammatory gene network analysis, including the four levels of COVID-19 severity (mild, moderate, severe, critical). We first estimate RNA and protein networks based on expression levels of RNA and protein, respectively (Analysis 1). Then, the interplays between RNA and protein networks are estimated via ligand–receptor pairs.

## 2. Results

We describe the results based on two parts: (1) characteristics of RNA and protein networks, and (2) interplays between RNA and protein networks. The characteristics of the networks are described by hub genes and COVID-19 severity-specific molecular interplays. Uncovering the mechanisms underlying COVID-19 severity is a crucial way to provide important insights for COVID-19 therapy; thus, we also focus on describing both severity-specific interplays and interplays between the RNA and protein inflammation networks.

The results can be briefly summarized as follows. The key finding from our inflammatory network analysis of COVID-19 samples is that ACKR2 plays a vital role, acting as a crucial broker between the RNA and protein inflammation networks. Our analysis reveals that AKCR2 facilitates protein interactions between the CCL family that affect the network of *S100A8*, which is the largest hub gene in the RNA network. In other words, the RNA network of inflammatory response genes is regulated by the protein network of the CCL family through AKCR2 acting as a broker. In other words, the protein network comprising the CCL family (ligands) interacts with ACKR2 (receptor) via ligand–receptor pairs (i.e., CCL family → ACKR2), then ACKR2 regulates the hub gene (S100A8) of the RNA network (i.e., ACKR2 → S100A8).

Our network analysis also shows that the inflammatory network is altered as early as the first stages of severity (mild phase), with these altered structures maintained as severity progresses.

### 2.1. Characteristics of Inflammation Networks Estimated from RNA and Protein Expressions

The total network data of RNA and protein expressions are provided in the Supplementary Information. Table 1 shows the total number of edges (i.e., the numbers of genes that are directly connected with the gene/protein) and the 20 top-ranked hub genes in each of the eight networks estimated from the above expression data.

Based on these data, a landscape of hubs was built in relation to COVID-19 severity and infection.

#### 2.1.1. Hubness of TOLLIP and TNFRSF4 in RNA Network and AGER and GHRL in Protein Network

Common hubs in the RNA inflammation networks based on the four groups of COVID-19 severity included TOLLIP (toll-interacting protein) and TNFRSF4 (tumour necrosis factor receptor superfamily member 4). On the other hand, AGER (advanced glycosylation end-product-specific receptor) and GHRL (ghrelin/obestatin prepropeptide) were common hubs in the protein inflammation network. In other words, hub genes/proteins play key roles in maintaining the inflammation networks; they connect and regulate many other genes within the networks, acting as central nodes. Such hubs are considered crucial biomarkers in gene network analysis, as they play pivotal roles in gene regulation and related biological processes. This suggests that TOLLIP, TNFRSF4, AGER, and GHRL are crucial inflammatory COVID-19 markers.

#### 2.1.2. High-Severity Specific Characteristics: Hubness of IL Family

The IL gene family (IL22RA1/IL20RB/IL17D in the RNA network and IL36A/IL20RB in the protein network) showed hubness for the moderate, severe, and critical samples; the average number of edges was 231.4 and 165.7, respectively, while the average number of edges for all genes/proteins in the RNA and protein networks was 95.0 and 92.6, respectively. This result indicates that higher activity on the part of the IL family can be considered a signature of severe COVID-19 samples. IL22RA1 has been identified as a crucial gene for critical COVID-19 and as a significant pathway involving viral protein interactions with cytokines, cytokine receptors such as IL22RA1 and TNFRSF10B, and cytokine–cytokine receptor interactions [16]. IL22RA1 is a crucial marker, with elevated concentrations associated with increased risk of death in cases of COVID-19. In previous studies, it has been recognized as a predictive marker of death in COVID-19 [17]. A previous study showed that the systemic concentrations of neutrophil chemokines (CXCL1/8), myeloperoxidase levels, and nasal IL17D transcription are correlated with the functional severity of COVID-19 [18].

Controlling for other variables such as age, disease severity, and ventilatory support, George et al. [18] also uncovered that IL-17C plasma levels remained independently associated with interstitial lung changes following COVID-19. Its association with disease progression in non-COVID-19 contexts such as fibrosing interstitial lung diseases further highlights the significance of this cytokine pathway in the development of fibrosis [18].

#### 2.1.3. Gene Ontology Enrichment Analysis of Hub Genes Provides Clear Distinction of Molecular Interplays

To identify the biological pathways and functions of the hub genes, gene enrichment analysis was performed using the DAVID bioinformatics tool (Database for Annotation, Visualization and Integrated Discovery). DAVID is a web server for functional enrichment analysis and functional annotation of gene lists. Gene ontology (GO) term annotations for the identified hub genes were performed using the categories “Molecular Function”, “Cellular Component”, and “Biological Processes” based on species and databases [19]. The hub genes are entered as a gene list on DAVID, with the 839 inflammatory response genes used as background genes.

A significance level of α=0.05 was considered, and GO terms corresponding to *p*-value <0.05 were considered as significant pathways. Table 2 shows the significant GO terms for the hub genes.

“GO:0004896 cytokine receptor activity” was a commonly over-represented GO term for the hub genes in the RNA inflammation networks of severe and critical samples. “GO:0004896 cytokine receptor activity” was identified as a top 10 most enriched GO term for the key common genes between COVID-19 and pericarditis [20]. Qi et al. [21] identified “GO:0004896 cytokine receptor activity” as a GO term associated with differentially expressed genes (DEGs) between patients with COVID-19 and normal controls. IL22RA1 and IL20RB were enriched in the severe and critical sample-specific GO terms. For protein networks, “GO:0007155 cell adhesion” was identified as a common GO term of the hub genes in the protein inflammation networks of the moderate, severe, and critical samples. Vastrad et al. [22] reported that “GO:0007155 cell adhesion” was among the enriched GO terms for the downregulated DEGs in SARS-CoV-2-infected samples compared to normal controls. This gene ontology enrichment analysis suggests that the hub genes of mild samples exhibit distinct molecular interplay compared to the samples from higher (moderate, severe, and critical) severity levels.

### 2.2. Uncovering Interplays Between RNA and Protein Networks via Ligand–Receptor Pairs

**Figure 2:**
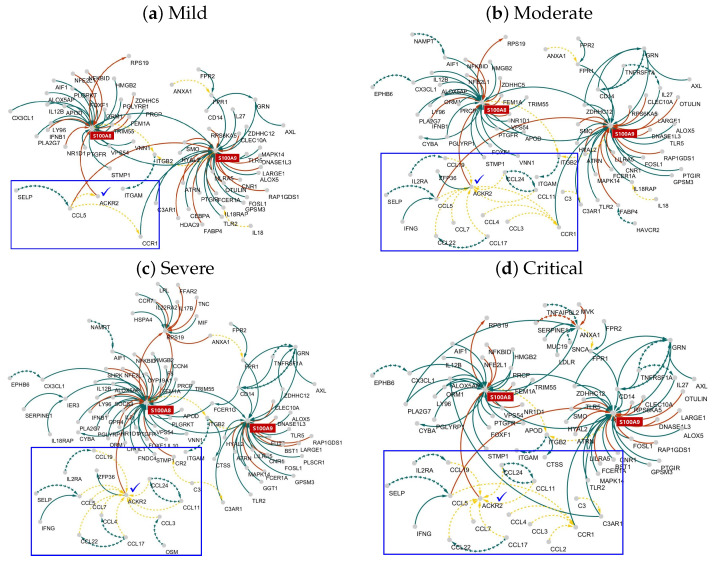
RNA and protein inflammation networks based on ligand–receptor pair; only the top 1% of edges are visualized. Each RNA (solid line) and protein (dashed line) network is estimated using the elastic network based on the RNA and protein expression levels, respectively, after which interplays between the two networks are extracted based on the ligand–receptor pairs. The line colors indicate types of interaction (green = activator, red = inhibitor, yellow = ligand–receptor pair). The blue box indicates interplays between RNA and protein networks via the broker gene ACKR2. Figures in (**a**–**d**) indicate the RNA and protein inflammation networks for mild, moderate, severe, and critical samples, respectively.

As seen in Table 1, several ten thousand edges cross over among several hundred genes/proteins in the networks. Weights (positive or negative values) are assigned to these edges. We retrieved the most essential subnetworks by selecting 1% of the edges for which the absolute values of the edge weights were in the top 1%. This procedure presented S100A8/S100A9 as the largest hubs in the networks of the four severity groups. Based on these results, we focused on the interplay between the hub genes S100A8/S100A9 and their neighborhood. The interactions between the RNA and protein inflammation networks were revealed using human ligand–receptor interaction pairs from CellTalkDB http://tcm.zju.edu.cn/celltalkdb/index.php (accessed on 25 March 2024). Ligand–receptor pairs were highlighted when the ligand (receptor) gene was present in the RNA network and the corresponding receptor (ligand) protein appeared in the protein network. Thus, RNA and protein inflammation networks were considered to be linked if ligand–receptor pairs existed between the networks. In order to effectively visualize these huge networks, we extracted 1% of the edges for which the absolute values of the estimated edge weights (i.e., ${\hat{\beta }}_{\ell j}$ in (2)) were in the largest 1%. Figure 2 shows the interplays between the RNA (solid line) and protein (dashed line) networks consisting of the extracted edges, where line colors indicate types of interaction (green = activator, red = inhibitor, yellow = ligand–receptor pair). The blue box indicates interplays between the RNA and protein networks mediated by ACKR2. The top right, top left, bottom right, and bottom left of Figure 2 indicate the RNA and protein inflammation networks for mild, moderate, severe, and critical samples, respectively. We focused on the neighborhood (i.e., children, grandchildren, parents, grandparents) of the markers, where a child node is a gene that is directly downstream of another gene in the network, meaning that it is regulated or influenced by a parent gene. In other words, a parent node is a gene that directly regulates or influences its child genes, and a grandparent node is two steps upstream in the regulatory path. This means that parent genes are often upstream regulators (e.g., transcription factors, receptors), while child genes are typically downstream responders. Thus, both parent and child genes are considered to be crucial nodes that control the gene regulatory system. The molecular interplay between S100A8/S100A9 and their neighborhoods as well as between ACKR2 and its ancestral genes (parents and grandparents) is shown in Figure 2, where the line colors indicate the type of edges (green = activator, red = inhibitor, yellow = ligand–receptor pair) and the solid (dashed) line indicates edges in the RNA (protein) network. The arrow indicates the direction from the regulator to the target (i.e., regulator → target genes). As shown in Figure 2a, the lowest severity levels (i.e., the mild samples) shows a distinct molecular interplay with the networks of moderate, severe, and critical samples (Figure 2b–d).

#### 2.2.1. Hubness of S100A8 and S100A9—Key Regulators

S100A8 and S100A9 show the highest hubness in the networks of the four COVID-19 severity groups, particularly the genes that are regulated by a large number of parent genes. Furthermore, S100A8 and S100A9 have similar regulatory structures in the networks of the four severity groups, with S100A8 and S100A9 having common parent genes and being regulated by same parent genes in the networks of mild, moderate, severe, and critical samples.

#### 2.2.2. Specific Characteristics of High-Severity Samples: S100A8 Regulates CYBA and CTSS

Although S100A8 and S100A9 have many parent genes, only a few of their child genes are identified. Table 3 lists the children and grandchildren of S100A8 and S100A9 in the gene network.

Because S100A8 and S100A9 regulate each other and have a large number of common child genes in the networks of the four severity groups, their grandparent genes through S100A8 and S100A9 are not considered. As shown in Table 3, RPS19, S100A9, and ITGB2 are common receptors of S100A8 in the networks of the four severity groups. In the networks of higher severity levels (moderate, severe, and critical samples), CYBA is a child of S100A8 and CTSS is a grandchild, while this interaction disappears in mild samples. Thus, the regulatory effect of S100A8 on CYBA and CTSS can be considered a characteristic of COVID-19 severity.

We identified S100A8 and CYBA as differentially expressed proteins between the non-susceptible and susceptible groups. Both of these have been implicated in the pathophysiology of severe COVID-19 and show increased expression in the non-susceptible group [23]. Ymam et al. [24] revealed that CTSS may function as a potential inflammatory biomarker that independently predicts unfavorable outcomes in individuals infected with SARS-CoV-2. CTSS is also clinically important for the prognosis of adult patients with COVID-19. CTSS has been observed to play a modest role in SARS-CoV infection, and may partially substitute for cathepsin L in certain cells [25].

#### 2.2.3. Specific Characteristics of High-Severity Samples: Ligand–Receptor Binding of CCL Family Between RNA and Protein Inflammation Networks

The CCL family contains ligand–receptor pairs with the parents of S100A8 and S100A9. In particular, the higher severity levels (moderate, severe, and critical) show their active interplay in the form of receptor–ligand binding between RNA and protein inflammation networks, whereas only receptor–ligand binding of CCL5 exists in the network of mild samples. The CCL family is a node in the protein network and a ligand of ACKR2, which is the parent of S100A8 in the RNA inflammation networks.

#### 2.2.4. Specific Characteristics of High-Severity Samples: ACKR2 Is a Crucial Broker Between RNA and Protein Inflammation Networks

Our multi-omics data analysis revealed a novel COVID-19 marker ACKR2 that acts as a crucial broker between RNA and protein inflammation networks. ACKR2 has interplay with a large number of CCL families via ligand–receptor pairing in the network of high-severity COVID-19 cases, as shown by the blue boxes in Figure 2. Furthermore, the molecular interplay between CCL family in the protein network can be considered a characteristic of severe COVID-19 infection. In summary, the protein network of the CCL family interacts with the RNA network of S100A8 via ACKR2. In other words, protein interactions via AKCR2 occur between the CCL family and the gene network of S100A, which is the largest hub gene in the RNA network. In particularly, the molecular interplay of ACKR2 is a characteristic of high COVID-19 infection levels, and its activity becomes weaker in the network of mild samples. Gomes et al. [26] discovered an increase in ILC2 cells expressing elevated levels of ACKR2 during the recovery phase, finding this to be correlated with serum markers associated with lung injury and repair. Furthermore, recovery from severe SARS-CoV-2 infection involves an expansion of ACKR2-expressing ILC2 cells, underscoring the importance of targeting this subset to promote lung recovery in COVID-19 and other related diseases [26]. Khalil et al. [27] revealed early upregulation of ACKR2 that remained stable during SARS-CoV-2 infection. Tveita et al. [28] revealed that ACKR2 levels tend to be elevated in COVID-19 patients relative to controls; moreover, they found similar increased levels in individuals infected with seasonal coronavirus or bacterial pneumonia. The cytokine/chemokine network plays a crucial role in initiating and sustaining the “cytokine storm”, a phenomenon that often leads to severe and potentially life-threatening COVID-19 disease progression [29].

**Figure 3:**
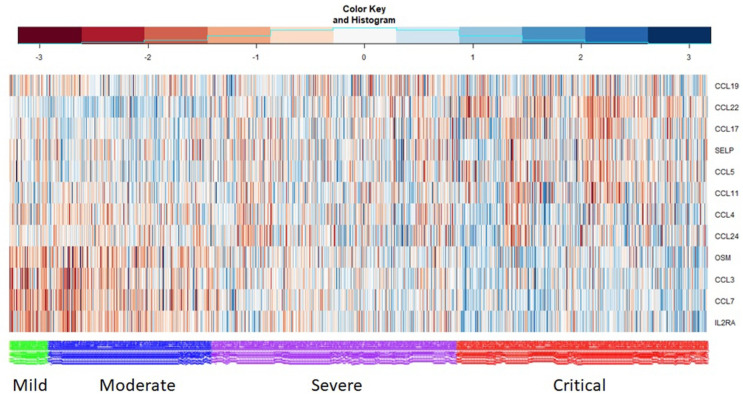
Heatmap of protein expression of the CCL family and its neighborhood (i.e., genes directly connected to the CCL family) in protein inflammation networks, where green = mild, blue = moderate, purple = severe, and red = critical. The *x*- and *y*-axes represent samples of COVID-19 severity stages and neighbor proteins of the CCL family, respectively. The histogram and color key in the columns presents the gene expression levels, with low levels (−3 to 0) shown in blue and high levels (0 to 3) in red.

Figure 3 shows the protein expression levels of the CCL family and its neighborhood (i.e., genes directly connected with the CCL family in protein inflammatory networks), where green = mild, blue = moderate, purple = severe, and red = critical.

The color in the heatmap presents low gene expression levels from −3 to 0 in blue and high gene expression levels from 0–3 in red. Genes in the subnetwork of the CCL family showed different expression levels in the four groups of COVID-19 severity; in particular, OSM, CCL3, CCL7, and IL2RA showed higher expression in the high-severity COVID-19 samples. The figure shows lower expression levels of CCL22 and CCL17 in high-severity COVID-19 samples compared to mild and moderate samples. This implies that molecular interactions between the CCL family and its neighborhood can represent crucial biomarkers for characterizing COVID-19 severity. Muri et al. [30] showed that elevated levels of specific chemokines (such as CCL2, CCL3, CCL4, CCL7, CCL8, CCL19, CXCL2, CXCL5, CXCL8, CXCL9, CXCL10, CXCL13, CXCL16, and CXCL17) are present in acute COVID-19 cases, and recent multi-omics studies have identified plasma chemokines as among the most influential factors associated with COVID-19 severity. According to Muri et al. [30], antibodies directed against COVID-19-associated markers such as CCL19, CCL22, and CXCL17 cluster together, and are sufficient on their own to correctly classify individuals as either uninfected controls or COVID-19 convalescents. According to Lu et al. [31], CCL2, CCL3, CCL5, and IP10 are critical initiators of the lethal immunopathological cascade in COVID-19. Peripheral blood levels of CCL5 were found to become elevated before IL-6 in severe COVID-19 patients. Furthermore, analyses using multi-omics techniques on bronchoalveolar lavage fluid from patients with COVID-19 have revealed signatures enriched with chemokines such as CCL2, CCL3, CCL4, CCL7, CCL8, CXCL2, CXCL8, CXCL17, and IP-10 [31]. In particular, CCL19 and CCL21 have been emphasized as markers of immune dysregulation in COVID-19, potentially indicating abnormal regulation induced by tissue inflammation [28].

These results imply that the interplay between ACKR2, the CCL family, and S100A8/9 can present vital information that aids in uncovering the complex mechanisms of COVID-19 severity. Accordingly, we suggest that ACKR2 is a crucial broker gene that links the RNA and protein inflammation networks. Thus, the interplay of the CCL family in the protein network regulates the molecular interplay of the S100A gene family in the RNA network via ACKR2.

#### 2.2.5. Summary of Specific Molecular Interplays in High-Severity Samples

Our gene network analysis reveals that the lowest (mild) level of severity displays a distinct difference in molecular interplay compared to higher-severity COVID-19 samples. The following COVID-19 severity-specific molecular interplays were identified in the higher-severity groups:

- ACKR2 is a crucial broker between the RNA and protein networks.
- The RNA and protein inflammation networks interact via ligand–receptor binding of the CCL family.
- CCL family interplay is present in the protein network.
- S100A8 regulates CYBA and CTSS.
- GO term of common hub genes include “GO:0004896 cytokine receptor activity” and “GO:0007155 cell adhesion”.
- The IL family shows active interplay.

These results suggest that effective control of severity-specific molecular interplay can provide crucial clues for uncovering the mechanisms of COVID-19 severity.

## 3. Data Source

### 3.1. RNA-Seq Data and Protein Expression Data

We analyzed the whole-blood RNA-seq data of 1102 genotyped samples obtained from the Japan COVID-19 Task Force. We considered four groups of COVID-19 severity: mild, moderate, severe, and critical. These levels of COVID-19 severity were defined as follows: critical (patients in intensive care unit or requiring intubation and ventilation); severe (patients requiring oxygen support); moderate (other symptomatic patients); and mild (without COVID-19-related symptoms) [11].

We also analyzed the protein expression (*n* = 2932 proteins from Olink Explore 3072) of 1384 genotyped samples consisting of 76 mild, 323 moderate, 487 severe, and 498 critical samples.

Table 4 shows demographic information for the samples used in the network analysis.

As shown in Table 4, older age groups (i.e., 50 or older) tend to have a higher ratio of severe samples. From a gender perspective, there is a greater proportion of males, and severe samples make up a large proportion of the overall samples.

**Figure 4:**
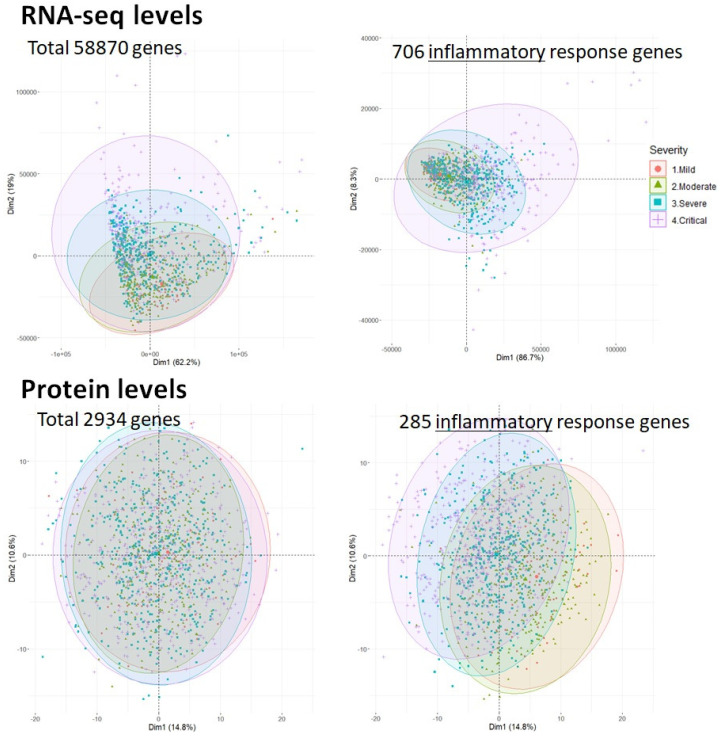
RNA and protein expression levels of four groups of COVID-19 severity (mild, moderate, severe, and critical) are shown in the first two principal component space, where the *x*- and *y*-axes indicate first and second principal components, respectively. The ellipses represent the 95% confidence intervals for each severity group. The top and bottom parts of the two figures indicate the expression levels of RNA and protein, respectively. At the top, the RNA expression of high severity levels also includes attributes present in the lower levels. In contrast, the expression levels of proteins are clustered into two groups: high (severe/critical) and low (mild/moderate). The colors of the contours indicate the severity level of COVID-19: red = mild, green = moderate, blue = severe, and purple = critical.

We focused on the 839 inflammatory response genes enriched in inflammatory response gene ontology (GO) terms (GO:0006954) and uncovered the molecular interplay between the inflammatory response genes. We extracted the expression levels of inflammatory response genes from the RNA and protein expression datasets. As a result, the RNA expression levels of 706 inflammatory response genes from 1019 samples and protein expression levels of 285 inflammatory response genes from 1384 samples were used in the network analysis. Figure 4 shows the expression levels of the four severity groups of COVID-19 cases in the first two principal component spaces (top: RNA expression levels; bottom: protein expression levels).

As shown at the top of Figure 4, the RNA expression of higher severity levels includes attributes present in lower levels. In contrast, the expression levels of proteins are clustered into two groups: high (severe/critical) and low (mild/moderate). These results imply that RNA and protein expression levels show different features in describing COVID-19 severity, and that the protein expression levels show characteristics that vary according to the COVID-19 severity phases.

### 3.2. Networks Estimated for Mild/Moderate/Severe/Critical Phases to Analyze the Interplays

For the 706 inflammatory response genes in RNA expression dataset, we estimated gene networks for the four groups of COVID-19 severity based on 71 mild, 241 moderate, 404 severe, and 303 critical samples. The gene networks of 285 inflammatory response genes were also estimated based on the protein expression levels of 76 mild, 323 moderate, 487 severe and 498 critical samples. In order to estimate the gene networks, we considered a regression framework. Although nonlinear regression models can capture more complex relationships, these models can be difficult to interpret [32,33,34]. On the other hand, linear models are typically simpler than nonlinear models, meaning that they are easier to interpret and explain. In gene network analysis, both network estimation and interpreting the estimated gene network represent crucial issues. Thus, many studies have performed gene network analysis based on linear regression models [35,36,37]. Here, we aim to uncover COVID-19 severity-specific interplays between inflammatory genes; as such, interpretation is a vital aspect of our study. Thus, we consider a linear regression framework rather than nonlinear regression and estimate the gene networks using an elastic net in which response and predictor variables represent the expression levels of target and regulator genes, respectively. The strength of the regulator genes with respect to the target gene (i.e., the edge weight) is described by the regression coefficient. The gene networks are estimated using the elastic net [38] technique. ELA is a widely used statistical approach for estimating gene networks in biomedical studies [39,40] because the elastic net can simultaneously perform edge selection and edge weight estimation. Here, we call the network estimated from RNA expressions the *RNA network,* while the network estimated from protein expressions is called the *protein network*. Each directed edge u→v in a network is assigned a weight representing the effect from *u* to *v* determined by the elastic net estimation based on the expression data. We estimate a total of eight networks, representing each of the mild, moderate, severe, and critical sample groups in terms of RNA and protein expression levels. The interplays between the RNA and protein networks are then described using ligand–receptor pairs.

## 4. Discussion

We analyzed the multi-omics data of COVID-19 samples obtained from the Japan COVID-19 Task Force. To reveal COVID-19 severity-specific inflammatory gene regulatory systems, we constructed gene networks based on the RNA and protein expression levels of inflammatory response genes for four COVID-19 severity categories (mild, moderate, severe, and critical). The identified common hub genes/proteins were strongly supported by previous studies as COVID-19 markers, and their mechanisms of action in SARS-CoV-2 and COVID-19 have been demonstrated. According to Li et al. [41], TOLLIP is a crucial marker in the NF-kB signaling pathway and presents a new focus for investigating COVID-19. Jin et al. [42] illustrated that SUMOylation limits the degradation of the ACE2 receptor via TOLLIP-mediated selective autophagy, thereby increasing host susceptibility to SARS-CoV-2, which could present a potential target for COVID-19 treatment. AGER plays a crucial role in COVID-19-induced lung inflammation, and the AGER pathway represents a therapeutic target for COVID-19 treatment [43]. In [43], the authors considered the similarity between the mechanistic principles that underlie a critical form of COVID-19 and the inflammation provoked by AGE and AGER interaction, along with the impact of diabetes mellitus on COVID-19 severity. Salehi et al. [44] investigated the involvement of several AGER ligands in triggering cellular inflammatory pathways. Elevated levels of AGER ligands such as AGEs, S100, and HMGB-1 have been found in COVID-19 patients with severe disease, and these levels were found to be significantly higher in patients with COVID-19 [45]. According to Waraich et al. [45], the upregulated AGER axis helps to elucidate the inflammatory and oxidative stress cascade observed in cases of severe COVID-19. Angioni et al. [46] reported that AGER acts as a functional receptor for SARS-CoV-2, thereby contributing to COVID-19 severity. The co-expression of TNFRSF4 and CD25 was identified in [47] as a better marker for measuring the SARS-CoV-2-specific helper T-cell response in COVID-19-vaccinated or convalescent individuals.

Our inflammatory gene network results showed differences between mild COVID-19 samples and higher-severity (moderate, severe, and critical) samples. The results also revealed the interplay between RNA and protein networks based on ligand–receptor pairs, with S100A8/9 showing the highest hubness in the networks of all four severity groups. The CCL family was also identified as a crucial marker of ligand–receptor binding between the RNA and protein inflammation networks. Furthermore, our analysis revealed that ACKR2 is a key broker of RNA and protein inflammation networks. ACKR2 is a receptor in the protein inflammation networks that is part of the CCL family. ACKR2 regulates S100A8, which was the hub gene with the largest hubness in the RNA inflammation networks. In particular, ACKR2 exhibited active interplay with its neighborhood in the high-severity (moderate, severe, and critical) samples, while the interplay became weaker in mild samples. Thus, ACKR2 plays a key role in the inflammatory gene networks of COVID-19 samples, and can be considered a crucial COVID-19 severity marker. ACKR2 does not rely on G-protein-coupled signaling, acting primarily as a scavenger receptor [48]. Its atypical signaling properties and ability to modulate chemokines likely allow it to mediate complex interactions between RNA and protein networks, with potential effects on immune regulation, inflammation, and tissue repair [49]. These properties can be seen as enabling ACKR2 to serve as a key mediator between the RNA and protein networks. In many previous studies, S100A8/A9 has been identified as a crucial marker for revealing the mechanism of COVID-19. Mellett et al. [50] suggested that S100A8/A9 could serve as a potential biomarker for COVID-19, as increased serum levels of S100A8/A9 are linked to severe COVID-19 cases and can differentiate between mild and severe disease. In individuals who tested positive for COVID-19, S100A8 expression was notably elevated relative to healthy individuals, reinforcing previous findings suggesting that S100A8/A9 can serve as a biomarker for assessing COVID-19 severity and predicting clinical outcomes [50]. S100A8 is significantly upregulated in both animal models and patients infected with SARS-CoV-2. Paquinimod is an S100A8/A9 inhibitor that has demonstrated potential in preventing COVID-19-associated immune disorders [51]. S100A8/A9 has been identified as an emerging biomarker for SARS-CoV-2 infection [52]. Bagheri et al. [53] suggested that S100A4, S100A9, and S100A10 are involved in inflammatory processes associated with COVID-19 and could potentially be used to predict disease severity. We suggest that suppressing severity-specific molecular interplays provides crucial clues to uncovering the mechanisms of COVID-19 severity. In particular, molecular interplay between ACKR2 and its neighborhood plays a key role in the inflammatory mechanism involved in COVID-19 severity. These results imply that ACKR2 could be considered a potential therapeutic target for assessing COVID-19 severity. Furthermore, S100A8 and S100A9 are also considered crucial therapeutic targets for inflammatory response to COVID-19. Thus, uncovering novel treatment approaches targeting the molecular system of ACKR2, S100A8, and S100A9 for COVID-19 is a future research step in our study.

Although this study is the largest to date for Asian COVID-19 genotyped samples, the male bias present in both the RNA (721 males vs. 298 females) and protein (987 males vs. 397 females) expression datasets must be considered as a limitation. The over-representation of male samples may bias the analysis towards male-specific biological signatures, particularly those related to disease severity and immune response. We will consider statistical correction methods to mitigate sex-based confounding as one of the future directions of this study.

The present study has identified severity-specific molecular interplays across RNA and protein inflammation networks using multi-omics data from COVID-19 patients. Notably, ACKR2 was found to act as a key broker linking protein-level CCL family interactions to the RNA hub gene S100A8, especially in high-severity cases. However, while this study presents important findings, an integrative synthesis that unifies these results into a therapeutic strategy for COVID-19 remains to be established. Future studies should aim to construct a systematic framework that contextualizes the observed molecular interactions within clinical disease trajectories. Investigations into the behavior of ACKR2 and S100A8/A9 expression and their modulation via therapeutic interventions may provide further insight into their roles as potential treatment targets. These directions will not only enhance the interpretability of the current findings but also guide the development of network-based therapeutic strategies for COVID-19 and related inflammatory conditions.

## 5. Method for Gene Network Estimation

Gene networks were represented using a linear regression framework. The expression levels of *p* regulator genes are provided as $X={\left(x_{1},\ldots ,x_{n}\right)}^{T}\in R^{n\times p}$, where $x_{i}=\left(x_{i1},\ldots ,x_{ip}\right)$, which control the $\ell^{th}$ target gene transcription $y_{\ell }\in R^{n}$ for *n* samples, ℓ=1,…,q. The following linear regression model was used to describe the gene regulatory system:

(1) $$\begin{array}{c} y_{i\ell }=\beta_{\ell }^{T}x_{i}+\epsilon_{i\ell },\;i=1,\ldots ,n,\;\ell =1,\ldots ,q \end{array}$$

where $\beta_{\ell }={\left(\beta_{\ell 1},\ldots ,\beta_{\ell p}\right)}^{T}$ is the regression coefficient vector that represents the effect of *p* regulator genes on the $\ell^{th}$ target gene and $\epsilon_{i\ell }$ is a random error vector.

An elastic net [38] was used to estimate the gene network:

(2) $$\begin{array}{c} \hat{\beta_{\ell }}=\underset{\beta_{\ell }}{\arg\;\min}\{\frac{1}{2}\sum_{i=1}^{n}{\left(y_{i\ell }-\beta_{\ell }^{T}x_{i}\right)}^{2}+\lambda \sum_{j=1}^{p}[\frac{1}{2}\left(1-\delta \right)\beta_{\ell j}^{2}+\delta |\beta_{\ell j}|]\},\;\ell =1,\ldots ,q \end{array}$$

where λ>0 is a regularization parameter that controls the degree of shrinkage applied to $\beta_{\ell }$ and 0≤δ≤1 is a mixing parameter between the ridge [54] and lasso [55] penalties. The regularization and mixing parameters were selected using cross-validation. In this study, the elastic net was implemented using the R package “glmnet” http://www.r-project.org.

## Figures and Tables

**Table 1:** Hub genes/proteins and total number of edges in the RNA and protein networks. The underlined items indicate common hubs in the networks of the four severity groups, with * indicating common hubs in the networks of higher-severity samples (i.e., moderate/severe/critical samples). The term “edge” indicates the numbers of genes directly connected to a specific gene/protein. A gene/protein with many connecting edges is considered to have broad effects on neighboring genes/proteins. Mild samples exhibit relatively sparse networks (i.e., small numbers of edges) in both RNA and protein inflammation networks compared with moderate, severe, and critical samples. Moderate, severe, and critical samples have many common hub genes, whereas mild samples show different molecular interplay structures in the inflammatory response networks.

	RNA				Protein			
Rank	Mild	Moderate	Severe	Critical	Mild	Moderate	Severe	Critical
1	BRD4	* IL22RA1	MEP1B	* PGLYRP2	PTN	* TLR2	* AHSG	* AHSG
2	TOLLIP	* PGLYRP2	GPR33	* IL20RB	IL1R1	* SMPDL3B	* TLR2	* SMPDL3B
3	CCL1	* IL20RB	* MSMP	* IL22RA1	RARRES2	* AHSG	GHRL	GHRL
4	PSEN1	CRHBP	CRHBP	CELA1	CXCL10	GHRL	* SMPDL3B	* TLR2
5	TICAM2	* TRIM55	* IL20RB	* MSMP	CRHBP	* NDST1	* IFNGR2	* IFNGR2
6	TUSC2	* CASP12	BDKRB2	ACKR2	CD200R1	* IFNGR2	* DPEP1	* NDST1
7	CLEC7A	* IL17D	* PGLYRP2	* CASP12	ATRN	PBXIP1	* NDST1	* DPEP1
8	P2RX7	SCN11A	* IL22RA1	CD5L	MIF	* DPEP1	PBXIP1	PBXIP1
9	PTGES	* MVK	* IL17D	* IL17D	SNAP23	* HMOX1	* IL20RB	* IL20RB
10	FFAR2	SPINK7	* CASP12	TOLLIP	BCR	* IL20RB	CD5L	SHPK
11	CD200	TOLLIP	* MVK	PRKCZ	PPBP	* ZP3	* VWF	* ZP3
12	CST7	PRKCZ	SMO	* TRIM55	PTPN6	AGER	* HMOX1	* VWF
13	TNFRSF4	ZEB2	ACKR2	CRLF2	GATA3	* VWF	* IL36A	* HMOX1
14	NFKBIZ	CLEC7A	CDH5	* MVK	MGLL	TLR3	AGER	FCER1A
15	PTGER3	* MSMP	TOLLIP	SMO	AGER	* EPHA2	ITGAV	TLR3
16	CERS6	TNFRSF4	FBXL2	KDM4D	VAMP8	NPY	* AGT	AGER
17	PXK	CD5L	SPINK7	RIPK1	IL1RAP	* AGT	* ZP3	SIRPA
18	STAP1	C1QTNF3	* TRIM55	TNFRSF4	TNFRSF1A	FCER1A	SIRPA	* EPHA2
19	ACKR2	RIPK1	TNFRSF4	CCL22	GHRL	* IL36A	* EPHA2	* IL36A
20	CCL26	F8	CRLF2	COL6A1	PARK7	ITGAV	REG3G	* AGT
♯ edges	23,922	36,447	39,024	34,755	6460	14,066	16,216	16,017

**Table 2:** Gene ontology enrichment analysis of the top 20 hub genes.

Network	Severity	Term	*p*. Value	Genes
RNA	Mild	Early endosome	0.047	PXK, TICAM2, TOLLIP, ACKR2
	Severe	Cytokine receptor activity	0.041	IL22RA1, IL20RB, CRLF2
	Critical	Cytokine receptor activity	0.041	IL22RA1, IL20RB, CRLF2
Protein	Mild	Extracellular region	0.001	IL1R1, RARRES2, PPBP,
				IL1RAP, PTN, MIF, AGER,
				TNFRSF1A, CXCL10,CRHBP,
				CD200R1, PTPN6, GHRL
		Schaffer collateral—CA1 synapse	0.047	BCR, GHRL, PTN
	Moderate	Extracellular matrix	0.012	VWF, AHSG, ZP3, TLR3
		Transmembrane signaling receptor activity	0.021	FCER1A, AGER, TLR3, TLR2
				IL36A, VWF, AHSG, NPY,
		Extracellular space	0.022	DPEP1, HMOX1, GHRL, ZP3
				SMPDL3B, AGT, TLR3
		Microglial cell activation	0.026	IFNGR2, AGER, TLR3, TLR2
		Cell adhesion	0.040	VWF, ITGAV, PBXIP1,
				AGER, EPHA2
	Severe	Cell adhesion	0.008	VWF, SIRPA, ITGAV, PBXIP1,
				AGER, EPHA2
		Cell migration	0.016	SIRPA, ITGAV, PBXIP1, EPHA2
				IL36A, VWF, AHSG, CD5L,
		Extracellular space	0.022	DPEP1, HMOX1, GHRL, REG3G,
				ZP3, SMPDL3B, AGT
	Critical	Extracellular matrix	0.012	VWF, AHSG, ZP3, TLR3
		Transmembrane signaling receptor activity	0.021	FCER1A, AGER, TLR3, TLR2
		Microglial cell activation	0.026	IFNGR2, AGER, TLR3, TLR2
		Cell adhesion	0.040	VWF, SIRPA, PBXIP1
				AGER, EPHA2

**Table 3:** Children, grandchildren, and grandparents of S100A8 and S100A9, where RNA, protein, and L-R indicate interactions in the RNA/protein inflammation networks and ligand–receptor pairs, respectively. The underlined items indicate high-severity markers.

		Mild		Moderate		Severe		Critical	
		Gene	Network	Gene	Network	Gene	Network	Gene	Network
S100A8	Child	RPS19	RNA	RPS19	RNA	RPS19	RNA	RPS19	RNA
		S100A9	RNA	S100A9	RNA	S100A9	RNA	S100A9	RNA
		ITGB2	L-R	ITGB2	L-R	ITGB2	L-R	ITGB2	L-R
				CYBA	RNA	CYBA	RNA	CYBA	RNA
	Grand child	S100A8	RNA	S100A8	RNA	S100A8	RNA	S100A8	RNA
		ITGAM	Protein	ITGAM	Protein	CTSS	RNA	CTSS	RNA
		ITGB2	L-R	ITGB2	L-R	S100A8	RNA	ITGAM	Protein
						ITGAM	Protein	ITGB2	L-R
						ITGB2	L-R		
	Grand parents	CCL5	LR	NAMPT	Protein	TNC	RNA	C3AR1	RNA
				EPHB6	Protein	FFAR2	RNA	LDLR	RNA
				CCL11	L-R	CCR7	RNA	MVK	RNA
				CCL17	L-R	IL17B	RNA	PLA2G7	RNA
				CCL19	L-R	ANXA1	RNA	MUC19	RNA
				CCL22	L-R	S100A8	RNA	SERPINE1	Protein
				CCL24	L-R	IL22RA2	RNA	EPHB6	Protein
				CCL7	L-R	HSPA4	RNA	TNFAIP8L2	Protein
						LPL	RNA	SNCA	Protein
						MIF	RNA	CCL11	L-R
						SERPINE1	RNA	CCL17	L-R
						IL18RAP	RNA	CCL19	L-R
						CD14	RNA	CCL22	L-R
						NAMPT	Protein	CCL24	L-R
						EPHB6	Protein	CCL7	L-R
						C3	L-R		
						CCL11	L-R		
						CCL17	L-R		
						CCL19	L-R		
						CCL22	L-R		
						CCL24	L-R		
						CCL3	L-R		
						CCL4	L-R		
						CCL5	L-R		
						CCL7	L-R		
S100A8	Child	S100A8	RNA	S100A8	RNA	S100A8	RNA	S100A8	RNA
		ITGB2	L-R	ITGB2	L-R	ITGB2	L-R	ITGB2	L-R
	Grand child	RPS19	RNA	CYBA	RNA	CYBA	RNA	CYBA	RNA
		S100A9	RNA	RPS19	RNA	RPS19	RNA	RPS19	RNA
		ITGAM	Protein	S100A9	RNA	CTSS	RNA	CTSS	RNA
		ITGB2	L-R	ITGAM	Protein	S100A9	RNA	S100A9	RNA
				ITGB2	L-R	ITGAM	Protein	ITGAM	Protein
						ITGB2	L-R	ITGB2	L-R
	Grand parents	CD14	RNA	CD14	RNA	CD14	RNA	CD14	RNA
		FPR2	RNA	GRN	RNA	GRN	RNA	GRN	RNA
		AXL	RNA	TNFRSF1A	RNA	TNFRSF1A	RNA	TNFRSF1A	RNA
		SELP	Protein	FPR2	RNA	FPR2	RNA	FPR2	RNA
		ANXA1	LR	IFNG	RNA	ZDHHC12	RNA	IFNG	RNA
		CCL5	LR	ZDHHC12	RNA	AXL	RNA	ZDHHC12	RNA
		IL18	LR	AXL	RNA	TNFRSF1A	Protein	AXL	RNA
				TNFRSF1A	Protein	GRN	Protein	TNFRSF1A	Protein
				SELP	Protein	ANXA1	L-R	SELP	Protein
				GRN	Protein	C3	L-R	TNFRSF1A	Protein
				HAVCR2	Protein			GRN	Protein
				ANXA1	L-R			ANXA1	L-R
				C3	L-R			C3	L-R
				CCL3	L-R			CCL2	L-R
				CCL4	L-R			CCL3	L-R
				CCL5	L-R			CCL4	L-R
				IL18	L-R			CCL5	L-R

**Table 4:** Demographic information of samples used in the network analysis.

		Age Group									Gender	
		20 s	30 s	40 s	50 s	60 s	70 s	80 s	90 s	100 s	Male	Female
RNA	Mild	7	16	7	4	8	12	13	4	0	33	38
	Moderate	29	48	52	34	51	21	6	0	0	162	79
	Severe	4	14	38	70	99	75	79	24	1	287	117
	Critical	0	6	20	54	86	75	52	10	0	239	64
Protein	Mild	7	17	7	4	8	16	13	4	0	35	41
	Moderate	35	55	60	45	62	34	24	8	0	218	105
	Severe	5	14	43	76	115	93	106	33	2	345	142
	Critical	1	8	28	78	140	137	92	14	0	389	109

## Data Availability

The RNA-seq expression and proteome data are available at the National Bioscience Database Center (NBDC) Human Database (accession code: hum0343; https://humandbs.biosciencedbc.jp/en/hum0343 (accessed on 2 March 2025)). Datasets of human ligand–receptor interaction pairs were obtained from CellTalkDB (http://tcm.zju.edu.cn/celltalkdb/index.php (accessed on 2 March 2025)).

## Supplementary Materials

The following supporting information can be downloaded at: https://www.mdpi.com/article/10.3390/ijms26094412/s1.
