# Supplementary figures and images for: Unraveling the COVID-19 Severity Hubs and Interplays in Inflammatory-Related RNA–Protein Networks
Source: Int J Mol Sci. 2025 May 6;26(9):4412. doi: 10.3390/ijms26094412 (PMC12072413; doi:10.3390/ijms26094412)

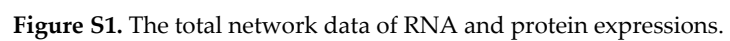

**Figure S1.** The total network data of RNA and protein expressions.

Supplement: Supplementary file 1 [file ijms-26-04412-s001.zip › ijms-3593690-supplementary.pdf]
